# Expectant Management of Miscarriage in View of NICE Guideline 154

**DOI:** 10.1155/2014/824527

**Published:** 2014-04-27

**Authors:** Junaid Rafi, Haroona Khalil

**Affiliations:** Ipswich Hospital NHS Trust Hospital, Heath Road Ipswich, IP4 5PD, UK

## Abstract

*Objective*. To find out the success rate of conservative management of complete two weeks for miscarriage in view of NICE Guideline 154. *Design*. Prospective observational study. *Setting*. Early pregnancy assessment units of District General Hospital in the United Kingdom. *Participants*. Women of less than 14 weeks' gestation, with a diagnosis of miscarriage (missed miscarriage/anembryonic or incomplete miscarriage). *Interventions.* Expectant management for two weeks. *Main Outcome Measure.* (1) Efficacy of 2-week expectant management, that is, complete resolution of miscarriage based either on self-reporting of patient after passing products of conception at home between D0 and D14 of expectant management or confirmation on scan at D14, and (2) short-term complications needing strong analgesia, blood transfusion, and antibiotics. *Results*. Expectant management of miscarriage for 2 weeks from the day of diagnosis was successful in 58% (64 /111) and failed in 42% (47/111). *Conclusions*. Expectant management success rate is consistent with the results from the longitudinal studies and RCTs published in the past. It is a safe option as none of the patients on expectant/medical management needed strong analgesia/antibiotics or blood transfusion.

## 1. Introduction

Approximately 11–15% [1] of pregnancies result in spontaneous first-trimester miscarriage and for some women, it could be quite traumatic experience physically as well as psychologically. Many women want to get over it and therefore are quite keen on active management; either medical or surgical; however, a sizeable percentage seems keen to explore conservative option of wait-and-see approach. The new NICE 154 Guideline recommends expectant management for 7–14 days as the first-line management strategy for miscarriage to explore management options other than expectant management if the woman is at increased risk of haemorrhage and had history of stillbirth, miscarriage, or ante partum haemorrhage in previous pregnancy; for example, a history of stillbirth, miscarriage or ante partum haemorrhage in previous pregnancy; coagulopathies; unable to have a blood transfusion or if there is evidence of infection. We aimed to find out the success rate of conservative management of complete two weeks for miscarriage.

## 2. Material and Method

We conducted this prospective longitudinal study from August 2012 to June 2013 in District General Hospital setting. The NICE Guideline 154 was published in December 2012; however, we started collecting data in our Early Pregnancy Assessment Unit (EPAU) for this study since August 2012 when the draft version of NICE Guideline 154 was available online. Over a ten months period, 130 cases were diagnosed as missed miscarriage, out of which 111 agreed for expectant management after counselling. We categorised miscarriage based on the ultrasound finding into missed miscarriage (MMC)/anembryonic pregnancy and incomplete miscarriage. For accuracy and uniformity, we calculated gestation by ultrasound measurements as well rather than from last menstrual period alone. We advised patients to inform us if they thought they had a completed miscarriage by noticing resolution of symptoms while being in the community before two weeks and were further instructed to do urine pregnancy test in two to three weeks' time. However if the process of miscarriage has not started or they had significant vaginal bleeding in that time period, a follow-up ultrasound scan was arranged in EPAU.

## 3. Outcome Measures

*(1) Expected Outcome*. The Outcome of expectant management was categorised as follows;

- complete resolution of vaginal bleed after passage of products at home: the patients were instructed “what to expect” (may notice clots with some tissue);
- negative pregnancy tests by the end of two weeks of conservative management if RPOC passed within this time.;
- we expected either no retained products of conception on ultrasound scan if patient is scheduled for the pelvic scan appointment on D14 or RPOCs less than 2 cm (no intervention was undertaken if there is no history of heavy bleeding).

*(2) Short-Term Complications*. Following were regarded as complication for the purpose of our study, that is, the need for strong (opiate) analgesia, evidence of infection requiring antibiotics, or heavy vaginal bleeding required blood transfusion due to hemodynamic instability.

## 4. Results

In this study, we found that 20% women were <25 years, 39% aged between 25 and 30 years, whereas 41% were 31–46 years of age. 45% were primiparous and multiparous found to be 55%. Our study revealed that 71% of (79/111) pregnancies were <9 weeks, whereas 27% (30/111) were 9–12 weeks of gestation and only 2 patients were found to be >12 weeks of gestation. The 95% cases were diagnosed as MMC and incomplete miscarriage in 5% cases based on ultrasound findings. After accepting conservative management for two weeks (which started from the day of ultrasound diagnosis of miscarriage), we found it to be successful in 58% (64/111) cases while being unsuccessful in 42% (47/111). In the successful group, 17% (11/64) were managed at community level that did not require a follow-up at EPAU or a scan. However 50 patients of this study attended follow-up to our EPAU and were triaged by assessments of symptoms and urine test for pregnancy. The patients with minimal symptoms and negative pregnancy test (22/64 = 34%) were discharged from EPAU without a scan. Therefore 51% of successful conservative management group did not require a pelvic scan. The 28 (43%) patients of this group had a scan, dictated by symptoms or positive test for pregnancy or both, were found to have complete miscarriage or RPOC less than 2 cm, and therefore as per our study protocol were discharged home. The three patients were admitted due to significant vaginal bleeding and passed RPOC in the hospital and later scan confirmed complete miscarriage.

**Figure 1:**
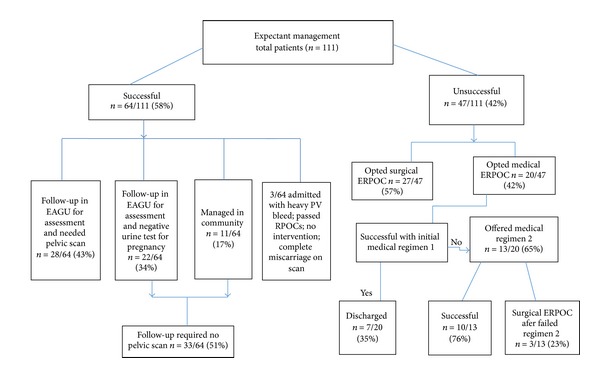
Result summary. Regimen 1: misoprostol loading dose of 800 mcg followed by 400 mcg per vagina after 6 hours. Regimen 2: further three doses of 400 mcg misoprostol (3 hours apart) per vagina if there is no response to regimen 1.

Out of the unsuccessful group, 27 patients opted for surgical management and the remaining 20/47 chose medical management. The patients who accepted medical management received 800 mcg of misoprostol as loading dose and 6 hours later had 400 mcg PV (regimen 1). The 35% (7/20) responded to it and passed products of conception. The remaining 65% (13/20) were offered further three PV doses of 400 mcg misoprostol 3 hours apart from each (regimen 2). However some patients (3/13) in the later group did not respond and as a result required surgical evacuation of RPOC. None of the patients on expectant/medical management in our study needed (opiate) analgesia/antibiotics or blood transfusion. All the patients attended their planned follow-up (Figure 1).

## 5. Discussion

Before the publication of NICE Guideline 154, we used to offer conservative, medical, or surgical management options to the patients after confirming the diagnosis of miscarriage assuming that all are equally effective and no one is superior to other as seen by a Cochrane review by Nanda et al. [2]. In that review, they concluded that expectant and surgical management are equally effective and the women preference should be taken into consideration.

It is arguable that recruitment of women for trial involving expectant management may not be easy because “wait-and-see approach” for one or two weeks sometimes is not an acceptable option for women after the diagnosis of miscarriage. There are few published RCTs [3, 4], that recruited women for comparison of expectant, medical, and surgical management of miscarriage. The number of patients recruited for expectant management in MIST trial [3] was 398/802 whereas Shelley et al. [4] managed to recruit 40 women in their trial.

As there are not many RCTs comparing expectant versus medical versus surgical treatment therefore,we have to rely on the available data from the observational studies which showed variable success rate with expectant management in incomplete miscarriage ranges from 79 to 96% [5] and 25 to 85% [6] in missed miscarriage. A longitudinal study conducted by P. Schwärzler et al. [7] reported 62% success rate after two weeks of expectant management of missed miscarriage or anembryonic pregnancy. In this study, 85/108 women opted for expectant management. A similarly longitudinal observational study by Casikar et al. [8] reported overall 61% (124/203) success rate.

The two weeks' time frame for the expectant or conservative management of miscarriage is also questionable as we can find reports in literature that relate higher success rate with longer duration up to 6–8 weeks [9]. In this context, the most recent prospective study published in November 2013 by Al-Ma'ani et al. (expectant versus surgical management) recruited 109 women in expectant group and quoted 81.4% success rate with 4-week wait [10].

In our study, the 58% success rate after two weeks of conservative management is not different from the above mentioned studies. As the criteria for ultrasound scan findings of incomplete miscarriage or the presence of retained products at follow-up vary widely therefore, it is understandable to expect different success rate reported in different studies [3]. In our study, both clinical and ultrasound findings were used for outcome measure. We can argue that the reported higher success rate in some studies is seen when follow-up is based on clinical findings rather than based on ultrasound [11].

It is a common observation that anxiety of emotional or psychological stress is associated with miscarriage that may be more with “wait-and-see approach” in the absence of supportive counselling. Therefore we particularly emphasized this aspect while recruiting the patients in our study. There is no significant difference in pain, physical recovery, anxiety, or emotional disturbances between medical versus expectant management [12] and also for surgical versus expectant management [13].

## 6. Limitations

Though this was a noncomparative longitudinal study in which number of patients was relatively more as compared to many other observational studies published so far. Our follow-up rate was 100% and we believed that it was due to supportive counselling and a follow-up appointment on D14 with ultrasound scan was booked at the commencement of expectant management. However women were advised to report us if they had complete miscarriage with the resolution of symptoms during that two-week period. All outcomes of our study were documented in clinical notes. Our study showed good acceptance rate for conservative management. We believed that good counselling and explanation can overcome the problem of recruiting women for clinical trials or comparative studies for the management of miscarriage as faced by authors of various studies referred in the above text.

## 7. Conclusion

Our study showed that the expectant management success rate is consistent with the results published in longitudinal studies and RCTs conducted so far, before NICE Guideline 154 was available. It is a safe option with significant success rate and minimal requirement for opiate analgesia, antibiotics, or blood transfusion. The majority of the cases can be managed in community without the need for hospitalization. In our unit, it helped to establish nurse lead services and utilization of beds for elective gynaecology workload.

## Abbreviations

MMC: Missed miscarriage
EPAU: Early Pregnancy Assessment Unit
RCTs: Randomised control trials
RPOCs: Retained products of conception
ERPOC: Evacuation retained products of conception
PV: Per vagina.
